# Delayed gait recovery by resolution of limb-kinetic apraxia in a chronic hemiparetic stroke patient: A case report

**DOI:** 10.1097/MD.0000000000028711

**Published:** 2022-01-28

**Authors:** Sung Ho Jang, Dong Hyun Byun

**Affiliations:** Department of Physical Medicine and Rehabilitation, College of Medicine, Yeungnam University 317-1, Daemyungdong, Namku, Taegu, Republic of Korea.

**Keywords:** delayed gait recovery, diffusion tensor tractography, dopaminergic drugs, limb kinetic apraxia, stroke

## Abstract

**Rationale:**

This paper reports on a chronic hemiparetic stroke patient who showed delayed gait recovery due to resolution of limb-kinetic apraxia (LKA).

**Patient concerns:**

A 49-year-old man underwent comprehensive rehabilitation at a local rehabilitation hospital since 3 weeks after spontaneous intracerebral haemorrhage. However, he could not walk independently because of severe motor weakness in his right leg until 19 months after the onset.

**Diagnosis:**

At the beginning of rehabilitation at our hospital (19 months after onset), we thought that he had the neurological potential to walk independently because the unaffected (right) corticospinal tract and corticoreticulospinal tract were closely related to the gait potential, representing intact integrities. As a result, we assumed that the severe motor weakness in the right leg was mainly ascribed to LKA.

**Interventions:**

At our hospital, he underwent comprehensive rehabilitation including increased doses of dopaminergic drugs (pramipexole, ropinirole, amantadine, and carbidopa/levodopa).

**Outcomes:**

After 10 days to our hospital, he could walk independently on an even floor with verbal supervision, concurrent with motor recovery of the right leg. After 24 days after hospital admission, he could walk independently on an even floor.

**Lessons:**

We believe that the resolution of LKA in his right leg by the administration of adequate doses of dopaminergic drugs was the main reason for the delayed gait recovery in this patient. The results suggest the importance of detecting the neurological potential for gait ability of a stroke patient who cannot walk after the gait recovery phase and the causes of gait inability for individual patients.

## 1 Introduction

Gait dysfunction is one of the most common, disabling sequelae of stroke. Generally, 70% to 80% of stroke patients can eventually walk during the gait recovery phase, which is within 3 to 6 months of stroke onset. The remaining 20% to 30% of stroke patients do not eventually recover their ability to walk.^[1–3]^ Gait function is more likely to recover than hand function because leg motor function is less dependent on the lateral corticospinal tract (CST) than hand function.^[4–9]^ As a result, detailed knowledge of gait could aid in the recovery of gait ability in stroke patients.^[10,11]^ Gait requires coordination of various brain functions related to motor sensory, execution, attention, and visuospatial functions to achieve stepping movements, adequate leg and trunk muscle power and tone, maintenance of equilibrium, and interlimb coordination.^[1,12–14]^ Recent studies have reported that stroke patients regained their gait ability after the gait recovery phase of stroke.^[10,11,15–17]^ These studies suggested that delayed gait recovery was achieved by the recovery of motor weakness, relief of severe spasticity and joint contracture, control of truncal ataxia, resolution of limb-kinetic apraxia (LKA), or improvement of postural control.^[10,11,15–17]^ Therefore, individualised rehabilitation based on a precise analysis of the causes of gait inability in stroke patients who cannot walk during the gait recovery phase of stroke is necessary.

Limb-kinetic apraxia (LKA), a type of apraxia, refers to the inability to perform precise and voluntary movements of the extremities resulting from an injury to the premotor cortex or the corticofugal tract of the premotor cortex.^[11,18–21]^ The detection of LKA is clinically important because it can be resolved by the administration of dopaminergic drugs.^[11,22–26]^ On the other hand, the precise detection of LKA could be difficult because it is based on clinical observations of movement patterns (awkward, clumsy, coarse, and mutilated pattern of execution of simple movements) without specific assessment tools.^[11,18–21]^ Furthermore, detection can be more difficult in cases of severe weakness, because clinicians cannot observe the characteristic movement patterns of LKA.^[26]^ Several studies have reported patients who showed motor recovery by the resolution of LKA in patients with brain injury.^[11,22–26]^ In contrast, little is known about delayed gait recovery due to LKA resolution.^[11]^

This paper reports on a patient with chronic hemiparetic stroke who showed delayed gait recovery with LKA resolution.

## 2 Case report

**Figure 1:**
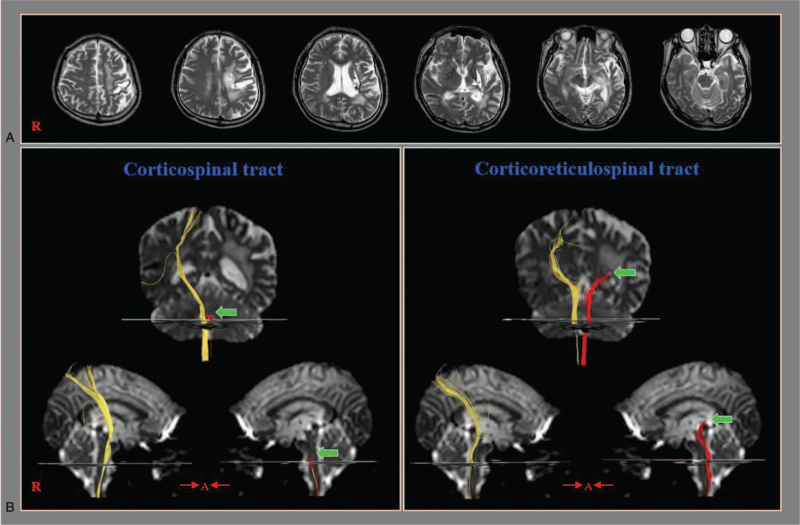
(A) T2-weighted brain MR images at 19 months after onset showing leukomalactic lesions in the fronto-parieto-temporal lobes and basal ganglia. (B) The left corticospinal tract and cortico-reticular-spinal tract show the discontinuations at the brainstem and subcortical white matter, respectively (arrows). In contrast, the right corticospinal tract and cortico-reticular-spinal tract reveal intact integrities.

A 49-year-old man underwent external ventricular drainage for spontaneous intracerebral haemorrhage (ICH) in the left basal ganglia at the neurosurgery department of a university hospital (Fig. 1A). Three weeks later, she was transferred to a local rehabilitation hospital. He was prescribed comprehensive rehabilitation, including dopaminergic drugs (pramipexole 1 mg, ropinirole 1 mg, amantadine 100 mg, carbidopa/levodopa 25 mg/250 mg, and bromocriptine 3.75 mg) and antispastic drugs (tizanidine 3 mg; baclofen 30 mg) until 19 months after the onset of ICH. However, the patient could not walk independently. He was admitted to the rehabilitation department of the university hospital, where he underwent external ventricular drainage 19 months after the onset. The Mini-Mental State Examination score was 25 (cut-off score <25).^[27]^ He had the following problems for an independent gait:

1. severe motor weakness of the right leg (manual muscle test [MMT]: hip flexor, trace; knee extensor, trace; ankle dorsiflexor, zero) and
2. severe spasticity of the right leg (Modified Ashworth Scale: knee flexor muscles, 3; ankle plantar-flexor muscles,
3. ^[28]^ He could not stand independently (Functional Ambulation Category [FAC]: 0 point, nonambulatory).^[29]^

To understand his neurological state, diffusion tensor imaging was performed, and 2 neural tracts (CST and cortico-reticulospinal tract [CRT]) were reconstructed using the diffusion tensor tractography reconstruction method.^[22,30]^ Although discontinuations were observed in the left (affected) CST and CRT, the unaffected (right) CST and CRT showed intact integrities (Fig. 1B). He underwent a rehabilitation program similar to the previous rehabilitation hospital: movement therapy at sections of physical and occupational therapy; at the bedside, motor strengthening of the trunk and left leg, exercises for trunk stability and control, static and dynamic balance training in sitting and standing positions, and neuromuscular electrical stimulation of the left knee extensor and ankle dorsiflexor muscles. In addition, the patient was given increased doses of dopaminergic drugs for recovery of motor function of the right leg (pramipexole 1->1.5 mg, ropinirole 1->1.5 mg, amantadine 100->150 mg, carbidopa/levodopa 25 mg/250 mg->50 mg/500 mg) and antispastic drugs for relieving severe spasticity of the right leg (baclofen: 30->50 mg and tizanidine: 3->4 mg).^[11,22–26,31]^ Ten days after hospital admission, he could walk independently on an even floor with verbal supervision (FAC: 3 points) concurrent with motor recovery of the right leg (MMT: hip extensor, poor ^-^[range: 20°]; knee extensor, poor; ankle dorsiflexor; zero) and relieving spasticity of the right leg (Modified Ashworth Scale: the knee flexor muscles, 2; ankle plantar-flexor muscles, 2). After 24 days of hospital admission, he could walk independently on an even floor (FAC: 3.5 points) with the recovery of motor weakness of the right leg (MMT: hip extensor, poor; knee extensor, fair; ankle dorsiflexor, zero). The patient provided written informed consent, and the institutional research board of the university hospital approved the study protocol.

## 3 Discussion

This paper reports a stroke patient who showed delayed gait recovery after approximately 1 month of rehabilitation, which started 19 months after ICH onset. Although the patient underwent rehabilitation with a similar program at the hospital for 18 months before admission to the hospital, he could not stand independently. At the beginning of rehabilitation at the hospital, the patient had the neurological potential to walk independently because the unaffected (right) CST and CRT are closely related to the gait potential, which represents intact integrities, even though the affected (left) CST and CRT revealed complete discontinuation.^[4–9]^ The following main problems were found to be responsible for his inability to walk:

1. severe motor weakness of the right leg and
2. severe spasticity of the right leg.

More dopaminergic drugs for motor recovery and antispastic drugs for relieving severe spasticity have been prescribed. After 24 days of rehabilitation at the hospital, the patient could walk independently on an even floor (FAC: 3.5). The resolution of LKA of the right leg was the main reason for the delayed gait recovery in this patient, although relieving the severe spasticity of the right leg also contributed to gait recovery for the following reasons. First, he revealed rapid motor recovery of the right leg by increasing dopaminergic drugs from 19 months after onset (knee extensor: trace (admission) -> poor (10 days after admission -> fair [24 days after admission]). This delayed rapid motor recovery of the affected leg was attributed not to motor recovery by neurological recovery, but to the resolution of LKA.^[11,22–26]^ Previous studies have reported motor recovery due to the resolution of LKA following the administration of dopaminergic drugs. The patient was also administered dopaminergic drugs during admission to a previous rehabilitation hospital. On the other hand, the doses of dopaminergic drugs did not appear to be sufficient for resolving LKA compared to the higher doses of dopaminergic drugs prescribed in previous studies.^[11,22–26]^ Second, he could walk without collapse of the hip joint during the stance phase of gait, even though the affected hip extensor muscles did not show sufficient muscle power to maintain the hip joint during the stance phase on physical examination, which is additional evidence of LKA. This patient did not exhibit muscle power of the affected hip joint due to LKA, even though he had sufficient muscle power to maintain the hip joint during the stance phase of gait.^[11,18–21]^

Several studies have reported the effects of dopaminergic drugs on the resolution of LKA in patients with brain injury.^[11,22–26]^ Among these studies, 1 study reported a patient who showed delayed gait recovery concurrent with the recovery of the affected leg weakness by administering dopaminergic drugs for 3 months since 41 months after a traumatic brain injury.^[11]^ To the best of our knowledge, this is the first study to demonstrate delayed gait recovery by resolution of LKA in patients with stroke. However, the generalisability of this study is limited because it is a single case report. Further studies will be needed, including a larger number of stroke patients who could not walk after the gait recovery phase. In addition, studies on the optimal doses for the resolution of LKA are necessary.

In conclusion, this paper reported a patient with chronic hemiparetic stroke who regained his ability to walk even at a chronic stage after the gait recovery phase by the resolution of LKA. The results suggest the importance of detecting the neurological potential for gait ability of a stroke patient who cannot walk after the gait recovery phase and the causes of gait inability for individual patients. In addition, administration of adequate doses of dopaminergic drugs is necessary when LKA is suspected.

## Author contributions

**Conceptualization:** Sung Ho Jang, Dong Hyun Byun.

**Data curation:** Dong Hyun Byun.

**Supervision:** Sung Ho Jang.

**Writing – original draft:** Sung Ho Jang, Dong Hyun Byun.

**Writing – review & editing:** Sung Ho Jang, Dong Hyun Byun.
